# Exploring the barriers to and facilitators of implementing CanRisk in primary care: a qualitative thematic framework analysis

**DOI:** 10.3399/BJGP.2022.0643

**Published:** 2023-06-13

**Authors:** Stephanie Archer*, Francisca Stutzin Donoso*, Tim Carver, Adelaide Yue, Alex P Cunningham, Lorenzo Ficorella, Marc Tischkowitz, Douglas F Easton, Antonis C Antoniou, Jon Emery, Juliet Usher-Smith, Fiona M Walter

**Affiliations:** Department of Public Health and Primary Care, Department of Psychology;; Department of Public Health and Primary Care;; Department of Public Health and Primary Care;; Department of Public Health and Primary Care;; Department of Public Health and Primary Care;; Department of Public Health and Primary Care;; Department of Medical Genetics, National Institute for Health Research Cambridge Biomedical Research Centre;; Department of Public Health and Primary Care;; Department of Public Health and Primary Care, University of Cambridge, Cambridge;; Department of General Practice and Centre for Cancer Research, University of Melbourne, Melbourne, Australia;; Department of Public Health and Primary Care, University of Cambridge, Cambridge, UK;; Wolfson Institute of Population Health, Barts and the London School of Medicine and Dentistry, Queen Mary University of London, UK.

**Keywords:** breast cancer, general practice physicians, implementation, multifactorial risk prediction, primary health care

## Abstract

**Background:**

The CanRisk tool enables the collection of risk factor information and calculation of estimated future breast cancer risks based on the multifactorial Breast and Ovarian Analysis of Disease Incidence and Carrier Estimation Algorithm (BOADICEA) model. Despite BOADICEA being recommended in National Institute for Health and Care Excellence (NICE) guidelines and CanRisk being freely available for use, the CanRisk tool has not yet been widely implemented in primary care.

**Aim:**

To explore the barriers to and facilitators of the implementation of the CanRisk tool in primary care.

**Design and setting:**

A multi-methods study was conducted with primary care practitioners (PCPs) in the East of England.

**Method:**

Participants used the CanRisk tool to complete two vignette-based case studies; semi-structured interviews gained feedback about the tool; and questionnaires collected demographic details and information about the structural characteristics of the practices.

**Results:**

Sixteen PCPs (eight GPs and eight nurses) completed the study. The main barriers to implementation included: time needed to complete the tool; competing priorities; IT infrastructure; and PCPs’ lack of confidence and knowledge to use the tool. Main facilitators included: easy navigation of the tool; its potential clinical impact; and the increasing availability of and expectation to use risk prediction tools.

**Conclusion:**

There is now a greater understanding of the barriers and facilitators that exist when using CanRisk in primary care. The study has highlighted that future implementation activities should focus on reducing the time needed to complete a CanRisk calculation, integrating the CanRisk tool into existing IT infrastructure, and identifying appropriate contexts in which to conduct a CanRisk calculation. PCPs may also benefit from information about cancer risk assessment and CanRisk-specific training.

## INTRODUCTION

Multifactorial cancer risk prediction models, such as the Breast and Ovarian Analysis of Disease Incidence and Carrier Estimation Algorithm (BOADICEA), have been shown to provide clinically valid risk estimates of developing breast cancer in the future.^1^^–^4 The use of the BOADICEA model is recommended in National Institute for Health and Care Excellence (NICE) guidelines.^5^ The BOADICEA model calculates a patient’s future risk of developing breast cancer by combining data on family history, demographic, lifestyle, and hormonal risk factors, pathogenic genetic variants, common genetic susceptibility variants, and mammographic density.^1^^,^^4^

In primary care, the NICE clinical guideline (CG164)^5^ currently recommends that a risk assessment, based on a first- and second-degree family history, should be performed when women with no personal history of breast cancer present with breast symptoms or concerns about relatives with breast cancer. The guideline also recommends the risk assessment in other clinically relevant consultations, such as women aged >35 years using an oral contraceptive pill or women being considered for long-term hormone replacement therapy (HRT) use. By using the BOADICEA model, the estimated risk of developing breast cancer — based on a multifactorial risk assessment — can inform women of their risk and identify those at above population-level risk who may benefit from referral to secondary care or to specialist genetics clinics.

**Figure 1.**
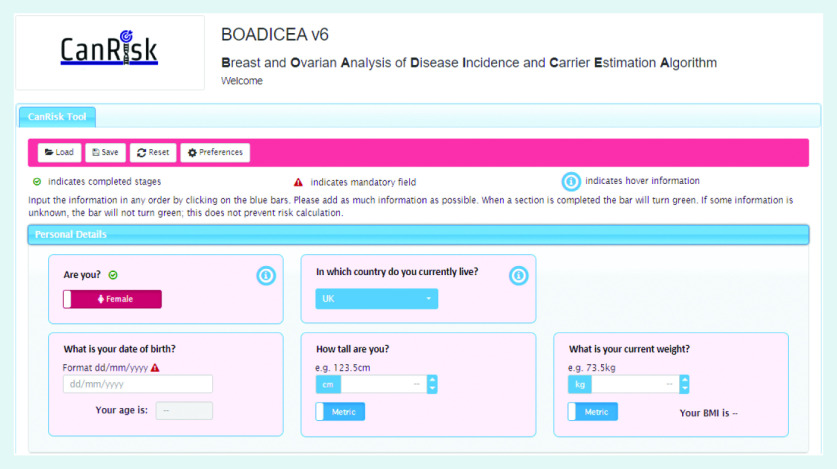
*Screenshot of the CanRisk tool showing how risk information is collected.*

**Figure 2.**
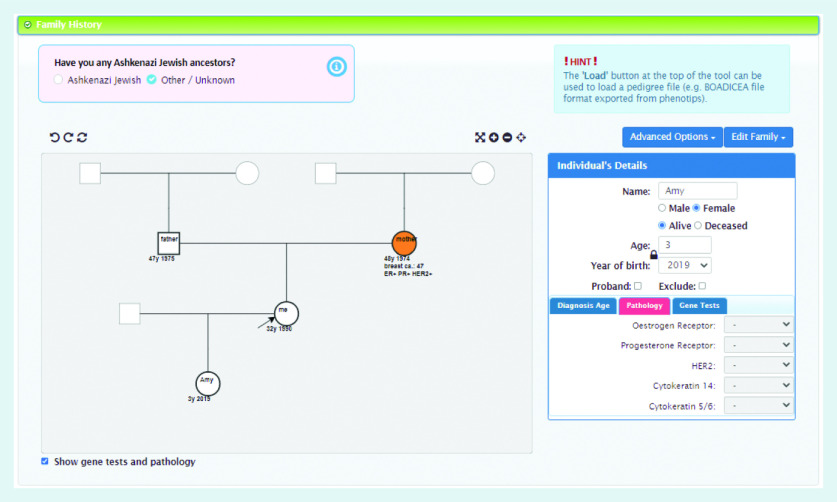
*Screenshot of the CanRisk tool showing how family history is collected.*

**Figure 3.**
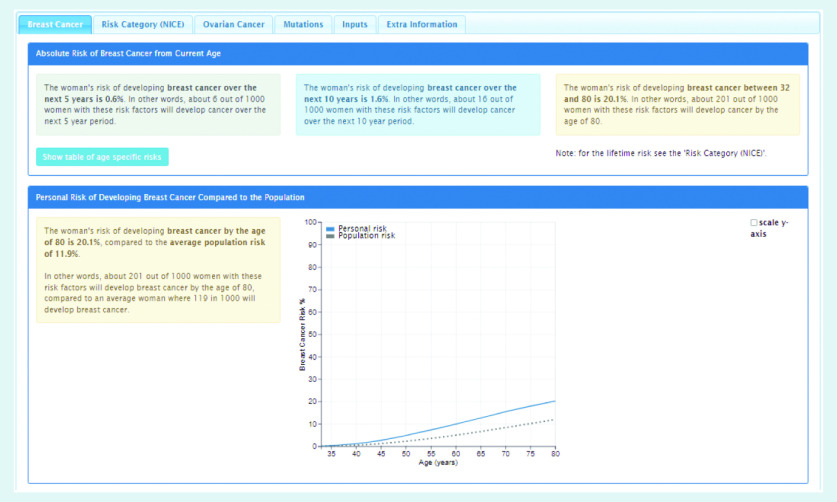
*Screenshot of the CanRisk tool showing how breast cancer risk is presented.*

In order to use the BOADICEA model in the clinical setting, a graphical user interface called the CanRisk tool (https://www.canrisk.org) has been developed to facilitate the collection of risk factor information (Figures 1 and 2) and provide healthcare practitioners with visual representations of risk to support effective communication (Figure 3).^6^ Developed for use by healthcare practitioners, the CanRisk tool has been shown to be both usable and acceptable in a variety of healthcare settings.^7^

**Table:** How this fits in

The CanRisk tool can help to identify women who are at increased risk of developing breast cancer. Although the CanRisk tool is used widely in specialist genetics clinics, it is not frequently used in primary care. The findings of the study, which emerged from talking to general practitioners and nurses, have enabled a better understanding of the barriers to and facilitators of using the CanRisk tool. How to better support GPs and nurses to use the CanRisk tool can now be planned.

More than 1 400 000 risk calculations have been conducted using the CanRisk tool since it received its CE marking (a legal requirement for medical devices being used in the European Economic Area) in 2020, with the majority of users coming from clinical genetics services (48%) or in secondary care (35%). Although primary care is the first point of contact for most women with concerns about their risk of breast cancer, <1% of CanRisk calculations in the UK are completed by GPs or practice nurses (PNs). This is despite GPs and PNs being well versed in discussing future risk of health conditions, and frequent users of digital risk prediction tools in a range of clinical areas.^8^

Several published studies have indicated why cancer-based risk prediction tools may not be widely used in primary care. In a survey of 400 UK-based GPs, >85% of participants reported that they had not heard of the two clinical prediction tools cited in the clinical guidelines at that time.^8^ Other studies have cited a range of personal and organisational barriers to the implementation of cancer risk assessment tools and cancer prevention more broadly.^9^^–^13 Personal barriers include the following: the role of clinical experience and belief in clinical intuition;^11^ a lack of knowledge of how to change patient behaviour around cancer prevention;^12^^,^^13^ and a lack of confidence to provide risk- reducing medication.^9^ Organisational barriers include the following: the need for additional funding;^10^^,^^13^ a lack of time available within the consultation;^9^^–^11^,^^13^ a lack of integration with the electronic health record (EHR);^11^ and the need for training.^9^^–^11^,^^13^

While many of these barriers may also apply to the implementation of the CanRisk tool, understanding the specific barriers and facilitators is important to i) inform further development of the CanRisk tool to increase its uptake in primary care; ii) inform the design of future CanRisk- based primary care studies; and iii) focus implementation activities on a local and national level. This study therefore aimed to explore the barriers to and facilitators of the implementation of the CanRisk tool in primary care and provide suggestions for future implementation activities.

## METHOD

### Design

This was a multi-methods study with the following three elements: 1) two clinical case studies; 2) a semi-structured interview; and 3) a questionnaire.

### Participants and recruitment

To gather the views of those most likely to use the CanRisk tool in clinical practice, GPs and nurses (including nurse practitioners [NPs] and advanced nurse practitioners [ANPs]) from the East of England were invited to take part in the study by the local Clinical Research Network. Practices were chosen if they could recruit at least one GP and one nurse. Data were collected between July and September 2020, during the first COVID-19 national lockdown.

### Data collection

A testing and interview session was conducted online; it was arranged at the participant’s convenience and lasted 1 hour. During the testing session, participants used the CanRisk tool to complete two clinical case studies presented in written vignettes (Box 1) while their screen was recorded. Following the testing session, participants were asked to share their thoughts and feedback through a semi- structured interview with a researcher with expertise in qualitative health services research. The interviews were designed to be short (<30 minutes), broad questions focused on the following: i) general feedback about the CanRisk tool; ii) their views on when and how the tool might be used in clinical practice; and iii) what (if any) factors might influence this (Supplementary Box S1). Participants were also asked to complete a questionnaire to collect their demographic information and to provide details about the structural characteristics of their practice.

**Box 1.** Example case study

**Summary**			
Annabelle Bates, a White British woman, has come to see you today to ask about changing her contraception. She has been on the oral contraceptive pill for 3 years, but her mother was recently diagnosed with breast cancer. She would like to know whether she is at high risk of breast cancer.			
**Age**: 32	**DOB**: 25/1/1990	**Height**: 1.63 m	**Weight**: 70 kg
**Lifestyle** Does not drink alcohol.			
**Women’s health** Had her first period when she was aged 9 years. Began taking the oral contraceptive pill 3 years ago.			
**Children** Has one child, Amy, who is aged 3 years.			
**Breast screening** Has never had a mammogram.			
**Medical history** Has no history of endometriosis. Has not had her tubes tied.			
**Family history**			
**Mother**: Annabelle’s mother is alive and is currently aged 48 years. She was diagnosed with breast cancer last year, aged 47. Her cancer was oestrogen receptor-positive, progesterone receptor-positive, and HER2- positive.			
**Father**: Annabelle’s father, who is 47 years, is well and has no history of cancer.			

### Data analysis

Video data from the two vignette- based case studies were used to inform improvements to the CanRisk tool. Qualitative data from the interviews were analysed using a deductive thematic framework approach,^14^ employing the constructs from the Inner Setting domain of the Consolidated Framework for Implementation Research (CFIR)^15^ (see Box 2 for an overview). The main reasons for this were as follows: 1) the intervention characteristics had already been assessed as part of the development process;^7^ 2) the semi-structured interview focused on barriers to and facilitators of hypothetically implementing CanRisk in participants’ specific practice; and 3) recruitment focused on two groups of clinical staff rather than other stakeholders (for example, patients, clinical commissioners, and policymakers).

**Box 2.** Constructs and subconstructs from the Inner Setting domain of the Consolidated Framework for Implementation Research (CFIR)^15^

**Structural characteristics**
The social architecture, age, maturity, and size of an organisation.
**Networks and communications**
The nature and quality of webs of social networks and the nature and quality of formal and informal communications within an organisation.
**Culture**
Norms, values, and basic assumptions of a given organisation.
**Implementation climate**
The absorptive capacity for change, shared receptivity of involved individuals to an intervention, and the extent to which use of that intervention will be rewarded, supported, and expected within their organisation. Subconstructs include: 1) tension for change; 2) compatibility; 3) relative priority; 4) organisational incentives and rewards; 5) goals and feedback; and 6) learning climate.
**Readiness for implementation**
Tangible and immediate indicators of organisational commitment to its decision to implement an intervention. Subconstructs include: 1) leadership engagement; 2) available resources; and 3) access to knowledge and information.

Four of the five CFIR Inner Setting domain constructs and their original definitions were used as the basis for the analytical framework and codebook (networks and communication; culture; implementation climate; and readiness for implementation). Two authors applied this framework to two transcripts and cross- checked the coding. Following this, one author coded the remainder of the transcripts using NVivo (version 12) and further refined the framework by generating subcategories. After completing the first round of coding, another author coded two transcripts with the revised framework to check for coding consistency. Information concerning the fifth CFIR Inner Setting domain (structural characteristics) was summarised from the questionnaire.

As the author leading the analysis had recently joined the CanRisk team, her distance from the project contrasted with the other author (who has a long standing investment in the overall CanRisk programme), which was valuable when conducting the analysis. In addition to this, the authors held frequent discussions regarding the interpretation of data and received feedback from the wider team.

## RESULTS

### Study participants

Sixteen primary care practitioners (PCPs; eight GPs and eight nurses [six PNs and two NPs]) from five general practices in the East of England took part in the study (Table 1). Participants had a mean age of 42.0 years (range 29–53) and 7.9 years (range 1–25) of clinical practice experience. Most participants (*n* = 15, 94%) felt either confident or very confident using a computer in their clinical practice, and (*n* = 14, 88%) used computer-based risk assessment tools in their patient consultations. None of the participants had experience with electronic pedigree family history drawing.

**Table 1.** Participant characteristics

**Characteristic**	**Total, *n* (%)^a^**	**GP, *n* (%)^a^**	**PN or NP, *n* (%)^a^**
**Participants**	16	8	8

**Age, mean, years (SD, range)**	42.0 (7.0, 29–53)	41.9 (6.9, 32–53)	42.1 (7.7, 29–52)

**Gender**			
Male	4 (25)	4 (50)	0
Female	12 (75)	4 (50)	8 (100)

**Years of experience, mean (SD, range)**	7.9 (6.9, 1–25)	10.0 (8.8, 2–25)	5.9 (3.9, 1–13)

**Level of confidence using computers in clinical practice**			
Not very confident	1 (6)	0	1 (13)
Confident	7 (44)	1 (13)	6 (75)
Very confident	8 (50)	7 (88)	1 (13)

**Current users of a computerised risk tool**			
Yes	14 (88)	8 (100)	6 (75)
No	2 (13)	0	2 (25)

**Experience using an electronic tool to draw family histories**			
Yes	0	0	0
No	16 (100)	8 (100)	8 (100)

a *Unless otherwise stated. NP = nurse practitioner. PN = practice nurse. SD = standard deviation.*

In line with the constructs within the Inner Setting domain of CFIR, information on the structural characteristics of the general practices is presented in Table 2. The data suggested that the practices were broadly comparable in terms of size, age, maturity, and social architecture. Some relevant features across practices include regular online or in-person staff training and regular clinical and administrative coordination meetings.

**Table 2.** Structural characteristics of research sites

**Site number**	**1** **[6 participants, 38% of sample]**	**2** **[3 participants, 19% of sample]**	**3** **[2 participants, 13% of sample]**	**4** **[2 participants, 13% of sample]**	**5** **[3 participants, 19% of sample]**
**Characteristic**					
**Patients registered, *n***	17 000	13 000	19 000	7000	9500
**Clinical staff, *n***	30	11	22	14	21
**Non-clinical staff, *n***	32	22	48	26	9
**Years practice opened for**	>55	>40	>25	>30	>30
**Electronic record system**	SystmOne	SystmOne	SystmOne	SystmOne	EMIS
**Staff training**	Training available through ‘Clarity Training’	Staff have mandatory training via website Opportunities for external training via monthly circular	Regular training. Much of it online via ‘Bluestream Academy’	Training available through ‘Bluestream Academy’ and the clinical commissioning group	Monthly training session for doctors Annual mandatory training
**Clinical coordination meetings**	Face-to-face weekly meetings	Partners meeting weekly that nurses can ask to attend if any pressing business Bi-monthly nurse meetings	Weekly lunchtime meeting	Monthly multidisciplinary team and clinical team meeting in-person and online (Microsoft Teams)	Bi-monthly staff meeting for all Monthly doctors’ meeting
**Admin organisation meetings**	Weekly meetings at practice level and monthly at Primary Care Network level (3 practices)	Regular reception meetings with PM and reception manager with nurse/and or GP input	At least once a month	Regularly in-person and online (Microsoft Teams)	Bi-monthly staff meeting for all Weekly meeting with PM, assistant PM, and senior partner discussing finance and human resource issues Daily huddle (15 minutes) for all working that day to discuss staffing, workload distribution across clinical and admin teams, plus any other relevant issues

*PM = practice manager.*

### Qualitative data analysis

The interview data mainly informed the constructs of implementation climate (59%) and readiness for implementation (40%). Networks and communication only gathered a small amount of data (<1%), and no data were coded under culture. Supplementary Table S1 presents the distribution of references across constructs and subconstructs.

### Implementation climate: compatibility

Interviewees reported that the integration of CanRisk into EHRs would simplify access and could facilitate the addition of results to patients’ records. Although full integration would be ideal to allow for data already recorded in the EHR to prepopulate the CanRisk tool, participants described accessing CanRisk via an external link as acceptable:

*‘But, as long as it’s integrated into SystmOne and can be saved in the notes that’s, honestly, the main thing, so as long as that happens it’s fine. And, as long as we can open it from SystmOne, like all the other tools we use, that’s fine, as well. So, as long as those two things apply the rest of it we can use as is.’*

(Male, GP, 15 years of experience, research site 2)

There was a general preference to collect the data required for the CanRisk calculation during patient consultations but, to save time, participants thought it would be helpful if patients could complete some information beforehand. Participants were aware of women’s potential emotional reactions to the risk information and saw consultations as the appropriate context to answer questions and offer support. As such, they expressed a preference for risk outcomes and management options being discussed in a consultation.

Appointments about oral contraception were cited as the most appropriate and relevant to complete CanRisk, followed by those about HRT, as breast cancer risk information is already part of these consultations. Attaching CanRisk to existing cervical screening (smear) appointments was also suggested, although some had concerns:

*‘Women are already often quite stressed when they come in for their smear and then to then without a warning shot say, oh, they leave the consultation saying, why did the nurse say that? Does that mean that I’ve got cancer … ?’*

(Female, GP, 3 years of experience, research site 1)

Most participants reported that all clinical staff would be able to collect the data to complete a CanRisk calculation owing to its usability. Furthermore, many reported that those who are directly involved with appointments about women’s health (for example, contraceptive and HRT appointments) would be able to communicate the risk result. However, there was some uncertainty as to who would be best suited to give management recommendations:

*‘I think in terms of the actual counselling element of it* […] *I think that our nurses who do contraceptive counselling should feel confident using this information. I suspect that most of them would then defer to a GP for* [a] *final decision, particularly regarding contraceptive changes.’*

(Female, GP, 3 years of experience, research site 1)

### Implementation climate: tension for change

Participants expressed that CanRisk would be a valuable tool in primary care. CanRisk was considered relatively easy to use, was similar to other computer-based risk assessment tools, and provided systematic and tangible information to reassure patients regarding their risk and offer them appropriate advice or management options:

*‘To just have something that you can just put in all the data and the figures, and come up with solid, sort of, evidence, and be able to say to the patient, well your risk is actually no greater than the general population. Again, that’s really useful, and I think that will be really reassuring for a lot of the patients that we see.’*

(Female, GP, 4 years of experience, research site 1)

Participants could see benefits in using CanRisk in the decision-making process for specialist services referrals of women who are worried about their breast cancer risk, or have a relevant family history of breast cancer:

*‘Because when we’re referring to genetics, a lot of the time we’ll be going: well, it sounds a bit far fetched and I’m sure it’s probably not necessarily an issue at all. But you are concerned, I don’t have the answer, therefore off you go.’*

(Male, GP, 7 years of experience, research site 1)

Despite the potential benefits of using CanRisk, participants also expressed worries about not all healthcare practitioners being as keen on using computer-based risk assessment tools and about the psychological impact that risk assessments can have on different patients:

*‘If the patient is a high risk and, you know, they’ve got all this medical diagnosis of anxiety, depression, panic attacks, I’ll be less inclined to be using this because even though it is clinically helpful and it will help them understand the risk, I feel like there’s still this other risk where they’ll take this home and then look at it and then be constantly worried about it.’*

(Female, GP, 2 years of experience, research site 1)

### Implementation climate: organisational incentives and rewards

Many participants described the importance of the tool being endorsed by the clinical commissioning group (CCG) and/or included in practice performance measures. Most of the participants who talked about this expressed that for PCPs to use CanRisk proactively, and not just in place of current discussions about family history of breast cancer, it would need to have funding attached and be somehow reimbursed. Examples of how this could be achieved are for CanRisk to be included in the Commissioning for Quality and Innovation (CQUIN) goals or the Quality and Outcomes Framework (QOF):

*‘Time, time is money, unfortunately. Which is very different for me coming from a background of secondary care then into primary care, where it’s, you know, it’s a business. I think, if there’s some incentive for it to be used, so whether it’s involved in some kind of QOF points or quality* […] *that would definitely, one hundred per cent, make it different.’*

(Female, NP, 3 years of experience, research site 5)

Furthermore, although CanRisk is currently free to use, any requirement for GP surgeries to pay for a licence to use it would negatively impact on its implementation.

### Implementation climate: goals and feedback

Beyond the financial aspects, participants also described the importance of giving visibility and support to new interventions for their uptake among PCPs:

*‘It’s just making sure people are aware of it I think, so cascading it and showing it to members of staff.’*

(Female, NP, 8 years of experience, research site 3)

Having the endorsement and promotion from commissioning services could help achieve this goal.

### Implementation climate: learning climate

Participants described already having dedicated time to learn about new interventions available and receive training on how to use them to increase their uptake:

*‘We have these clinical meetings on Tuesday afternoons* […] *if we are launching this, we’ve got this access to this then I’d probably take it in that meeting and show it to everyone how it’s used and then we just hope that everybody will use it.’*

(Female, GP, 20 years of experience, research site 3)

### Implementation climate: relative priority

Participants described how taking on new interventions and their associated training while continuing to deliver routine care can be overwhelming for PCPs, and even more so while adjusting and responding to the COVID-19 pandemic emergency:

*‘I think your biggest difficulty at the moment is, information overload. COVID has brought so many changes* […] *so much is coming, that I’m thinking, before, I’d love all this free education, but there’s only so much I can do.’*

(Male, GP, 25 years of experience, research site 4)

Still, participants thought that highlighting the potential clinical benefits of using CanRisk would help increase its relative priority:

*‘Rather than just sending patients to hospital, it’s like* […] *a very expensive service so if we can do something like that* [CanRisk]*, that will definitely help, yes, and it’s just, you can reassure the patient.’*

(Female, GP, 20 years of experience, research site 3)

### Readiness for implementation: available resources

As appointment length in UK primary care is limited (approximately 10 minutes), participants were concerned that they would need longer to complete CanRisk (which takes approximately 15 minutes) and discuss the outcomes with patients:

*‘Definitely have to extend the normal clinic time. I mean, if you could, you know, if you know the lady’s going to be low risk then that’s fine, isn’t it, you could quickly do it but I think you always have to account for a degree of counselling with the results so I’d definitely need to have extra appointment time.’*

(Female, PN, 6 years of experience, research site 5)

### Readiness for implementation: access to knowledge and information

Participants faced challenges when completing the family history section. They reported finding it difficult and time- consuming to complete as they had little experience of using pedigree drawing software. Participants expressed a desire for a simplified process with more prompts:

*‘So, you just need to keep it as quick and as simple as possible if you like. That bit at the end with the family history was tricky, I didn’t find that easy at all* […] *doesn’t always make it obvious where you have to click on things.’*

(Female, PN, 13 years of experience, research site 2)

Despite their experience completing risk assessment tools and discussing risk information with patients, both GPs and nurses (PNs and NPs) lacked confidence regarding their general breast cancer knowledge to discuss CanRisk outcomes and management options with patients:

*‘In terms of confidence, I wouldn’t say I’m very high up confident on that, but I think the good thing about being a GP is you can always say* […] *to the patient, I’m not sure, I’ll do my research and I’ll come back to you in a bit.’*

(Female, GP, 2 years of clinical experience, research site 1)

When thinking about how and when they might offer risk management options to women who are at moderate risk in the future, participants reported that they would lack the experience and confidence to prescribe risk-reducing medication, as this is currently initiated in secondary care and/or specialist genetics clinics:

*‘If a tool like this said so, I might do* [prescribe risk-reducing medication]*, yeah.* […] *had that situation been presented in real life, I might send an advice and guidance request to genetics or the breast clinic* […] *But no, I’ve never prescribed that before.’*

(Male, GP, 4 years of experience, research site 1)

Therefore, beyond learning about the CanRisk tool and how to navigate it, support on how to interpret and deliver CanRisk outcomes seems necessary for its implementation in primary care:

*‘I think to have some training to interpret the information would be good and because obviously it’s a really sensitive subject that you’re discussing, if somebody comes out at a really high risk and then to try to then explain it to them, that’s quite hard if you haven’t had any kind of training.’*

(Female, PN, 2 years of experience, research site 4)

### Networks and communication

Participants suggested that good team communication and wider networks would enable primary care users to support each other and increase their confidence:

*‘We have a very supportive sort of team as well, so if there’s something that I wasn’t sure then we have all the GPs and things here that we could sort of flag up concerns or anything. If there was something that was glaringly sort of, you know, risk factor wise, then obviously then we could sort of consult with* [GP] *as well.’*

(Female, PN, 1 year of experience, research site 1)

## DISCUSSION

### Summary

Most barriers to and facilitators of the implementation of the CanRisk tool in primary care clustered around the following: time needed to complete the CanRisk tool; the tool’s compatibility and integration with existing primary care IT systems and ways of working; competing priorities; and the need for training and capacity building within healthcare teams (Box 3).

**Box 3.** Clusters arising from the barriers to and facilitators of implementation

**Cluster**	**Barriers**	**Facilitators**
Time taken to complete the tool	Amount of time required to complete a CanRisk assessment, communicate the outcomes, and discuss management options.	The tool is easy to navigate by GPs and nurses (PNs and NPs). A patient-facing version of the CanRisk tool could facilitate risk factor and family history data capture.
Compatibility and integration with existing primary care IT systems and ways of working	Lack of integration of the CanRisk tool into the EHR. Individual resistance to electronic risk assessment tools.	Professionals are already used to similar risk assessment tools.
Opportunities for use within primary care	The competing priorities within primary care. Causing anxiety to patients.	The potential clinical impact of the CanRisk tool in general and its potential value in appointments (for example, pill checks, HRT, and smear tests) where discussions around breast cancer risk happen. The potential benefit of reassuring women in the population risk category.
Need for training and capacity building within healthcare teams	Professionals’ lack of confidence regarding their knowledge about breast cancer. Professionals’ lack of experience using electronic pedigree family history drawing tools. Time-consuming and complicated family history section in the current version of the CanRisk tool. Professionals’ lack of knowledge and experience prescribing risk-reducing medication.	General practices having protected training time to learn about new interventions such as the CanRisk tool. Relationships of trust and support among professionals within a given setting.

*EHR = electronic health record. HRT = hormone replacement therapy. NP = nurse practitioner. PN = practice nurse.*

### Strengths and limitations

Equal numbers of GPs and nurses (PNs and NPs) were interviewed who ranged in age and experience of clinical practice. As many of the structural characteristics of the practices were similar, and practices consenting to take part may have been more research active or have a particular interest in cancer, greater diversity in terms of location, practice size, and practice organisation, as well as sites that are non- research active, may add additional depth to the results in future studies. The interview schedule was broad and not based on the CFIR constructs as the interviews focused on participants’ experiences of using the tool through case studies and their general views on implementation were based on their clinical experience. A greater focus on CFIR during the data collection stage may have resulted in a more nuanced discussion of specific elements. This study focused on GPs and nurses, as they are the PCPs most likely to use the tool in clinical practice, and data were not collected from allied healthcare professionals (for example, healthcare assistants and practice pharmacists) or key stakeholders (for example, patients, commissioners, and policymakers) who would be able to give insight into other domains of CFIR.

### Comparison with existing literature

While the data from the interviews (further supported by videos from the vignette- based case studies) indicated that the CanRisk tool was considered accessible and easy to navigate, there were concerns about whether PCPs could complete a risk calculation and offer appropriate counselling to the patient within a typical UK primary care appointment (which is an average of 10.9 minutes).^16^ This is consistent with the authors’ previous work exploring the acceptability of the CanRisk tool in multiple settings and countries,^7^ as well as the broader body of literature around incorporating cancer risk assessment into practice.^9^^,^^10^^,^^17^^,^^18^ As a way of reducing the amount of time needed to complete a CanRisk calculation in clinic, the development of a patient-facing version of the tool received broad support.

While some of the data required to complete the CanRisk tool (for example, body mass index [BMI] and contraceptive use) may be easily entered into a patient- facing version of the tool, collecting information on family history is more challenging. Despite an established need for family history data collection tools,^19^^,^^20^ and several having been designed specifically for use in primary care^21^ and/or cancer care,^22^ even highly promising tools^23– 29^ are not currently routinely used in UK primary care, owing to the time they take to complete, the accuracy of information collected, and the need to update them over time. With these points in mind, any development of a patient-facing version of the CanRisk tool that includes family history should use the gold standard of user-centred design,^30^^,^^31^ take into account the elements of existing designs that have already been tested, and undertake appropriate evaluation and validation studies.

Consistent with previous research,^9^^,^^10^ the present study shows that integrating the CanRisk tool with existing NHS IT infrastructure is essential for successful implementation in primary care. There are important issues for complex digital multifactorial risk prediction tools, such as CanRisk, as the fragmented local governance structure would require the CanRisk tool to be approved and adopted by governance and IT teams on a service- by-service basis, or the BOADICEA model becoming integrated within existing EHRs (for example, EMIS or TTP) or via third-party providers (for example, Ardens). The integration and implementation of new technology within the NHS has traditionally been a complex and lengthy process. However, the transformation of digital services, following the unprecedented need to adapt during the COVID-19 pandemic, may be of benefit in facilitating the use of the CanRisk tool in primary care and sharing and updating the results within a range of services within the NHS.^32^^–^34

As in other studies, the participants in the present study described that primary care presents a clear opportunity for CanRisk to identify patients at increased risk of cancer who would benefit from interventions focusing on early detection and prevention.^10^^,^^35^ However, the potential benefits of using the CanRisk tool were juxtaposed to the increased effort in conducting the risk assessment, and receiving training on how to do so, in a setting where there appears to be an ever- increasing workload and rapidly dwindling resources.^36^^–^38 Several suggestions were put forward that focused on regular women’s health appointments (for example, contraceptive-pill checks and HRT appointments), which may be suitable as they provide opportunities to i) approach the topic of risk assessment as part of a wider conversation on cancer risks, and ii) easily talk about hormonal and lifestyle risk factors that may contribute to their risk score.^39^

The study identified that training and capacity building within healthcare teams is likely to have a significant role in the implementation of the CanRisk tool. The development of bespoke training resources around CanRisk is required but, for now, centralised genetics-focused training initiatives (for example, GeNotes^40^ and QGenome^41^) may be helpful in improving knowledge, skills, and attitudes around taking and understanding family histories. A lack of knowledge around breast cancer and a lack of experience of risk-reducing medication were also cited as potential barriers to implementation. While training on these topics was highly desirable, the participants were concerned about the competing demands in clinical practice, particularly following the COVID-19 pandemic. As such, any educational intervention designed to provide PCPs with the knowledge and skills to use the CanRisk tool should consider the best mode of delivery^42^^,^^43^ and if protected learning time is required.

### Implications for research and practice

Based on the findings from this study, future work to facilitate the implementation of the CanRisk tool in primary care should focus on four main areas. The first area is developing a user- friendly interface that allows patients to enter some of the risk factor information before a clinical appointment. The second is establishing a mechanism for the integration of the CanRisk tool within the EHRs used in the primary care setting. The third is exploring and testing which clinical appointments might be most appropriate for introducing and performing a CanRisk calculation. The final area is developing a training package to support healthcare practitioners to use the tool, interpret the findings, and make choices about the next steps following the risk assessment.

In conclusion, a range of barriers to the implementation of the CanRisk tool in primary care exist, predominantly focusing on the amount of time needed to complete the assessment using the tool, the need for integration with existing IT systems, realising the opportunity in the context of competing demands on PCPs’ time, and the need for training on a range of clinical topics. Future work to overcome these barriers will be prioritised following the recommendations presented here.

## Data

Data are unavailable as participant consent was not sought.
